# Interleukin 27 is a novel cytokine with anti-inflammatory effects against spondyloarthritis through the suppression of Th17 responses

**DOI:** 10.3389/fimmu.2022.1072420

**Published:** 2023-02-02

**Authors:** Quentin Jouhault, Bilade Cherqaoui, Aude Jobart-Malfait, Simon Glatigny, Marc Lauraine, Audrey Hulot, Guillaume Morelle, Benjamin Hagege, Kétia Ermoza, Ahmed El Marjou, Brigitte Izac, Benjamin Saintpierre, Franck Letourneur, Séverine Rémy, Ignacio Anegon, Marie-Christophe Boissier, Gilles Chiocchia, Maxime Breban, Luiza M. Araujo

**Affiliations:** ^1^ Infection & Inflammation, UMR 1173, Inserm, UVSQ/Université Paris Saclay, Montigny-le-Bretonneux, France; ^2^ Laboratoire d’Excellence Inflamex, Université Paris-Centre, Paris, France; ^3^ Plateforme de production d’anticorps et de protéines recombinantes-Institut Curie/CNRS UMR144, Paris, France; ^4^ Plateforme GenomIC- Université de Paris, Institut Cochin, INSERM-CNRS, Paris, France; ^5^ Platform Transgenic Rats and ImmunoPhenomics, INSERM UMR 1064-CRTI, Nantes, France; ^6^ Inserm UMR1125-Université Sorbonne Paris Nord, Rheumatology Division, Avicenne Hospital (AP-HP), Bobigny, France; ^7^ Haematology-Immunology Division, Ambroise Paré Hospital (AP-HP), Boulogne-Billancourt, France; ^8^ Rheumatology Division, Ambroise Paré Hospital (AP-HP), Boulogne-Billancourt, France

**Keywords:** spondyloarthritis, dendritic cell, HLA-B27, JAK, IL-27, IL-17, IL-10, animal models

## Abstract

**Introduction:**

Spondylarthritis (SpA) development in HLA-B27/human β2-microglobulin transgenic rat (B27-rat) is correlated with altered conventional dendritic cell (cDC) function that promotes an inflammatory pattern of CD4+T cells, including a biased expansion of pro-inflammatory Th_17_ population and imbalance of regulatory T cells cytokine profile. Transcriptomic analysis revealed that cDCs from B27-rats under express IL-27, an anti-inflammatory cytokine which induces the differentiation of IL-10^+^ regulatory T cells and inhibits Th_17_ cells.

**Methods:**

Here, we first investigated whether *in vitro* addition of exogenous IL-27 could reverse the inflammatory pattern observed in CD4^+^ T cells. Next, we performed preclinical assay using IL-27 to investigate whether *in vivo* treatment could prevent SpA development in B27-rats.

**Results:**

*in vitro* addition of IL-27 to cocultures of cDCs and CD4^+^ T cell subsets from B27-rats reduced IL-17 and enhanced IL-10 production by T cells. Likewise, IL-27 inhibited the production of IL-17 by CD4^+^ T cells from SpA patients. Interestingly, *in vivo* treatment with recombinant IL-27 starting before SpA onset, inhibited SpA development in B27-rats through the suppression of IL-17/TNF producing CD4^+^ T cells.

**Discussion:**

Overall, our results reveal a potent inhibitory effect of IL-27 and highlight this cytokine as a promising new therapeutic target in SpA, especially for SpA patients non responders to currently approved biotherapies.

## Introduction

Spondyloarthritis (SpA) is a group of chronic inflammatory disorders that primarily affect axial skeleton (i.e. spine and sacroiliac joints). This cardinal feature often combines with peripheral joint arthritis, enthesitis, dactylitis and specific extra-articular manifestations, i.e. uveitis, psoriasis or inflammatory bowel disease (1). It affects between 0.2% and 1.61%, of the population worldwide and is strongly associated with the class I major histocompatibility complex (MHC) antigen HLA-B27 (2, 3). This association was described almost 50 years ago but the mechanisms underlying induction of SpA by HLA-B27 still remain largely unexplained (4).

The 33-3 line of HLA-B27/human β2-microglobulin (hβ2m) transgenic rats (B27-rat) has proven to be a thorough model of SpA since it develops a spontaneous inflammatory disorder combining arthritis with ulcerative colitis and psoriatic skin and nail lesions, in the presence of regular microbiota (5). In this animal model, cell transfer experiments have demonstrated a fundamental role of bone-marrow derived myeloid cells expressing the HLA-B27/hβ2m transgene and CD4^+^ T cells in disease development (6, 7). Thus, further studies revealed a correlation between conventional dendritic cells (cDCs) dysfunctions and SpA susceptibility (8–10).

We previously showed that cDCs from B27-rat promoted a biased expansion of pro-inflammatory T helper 17 (Th_17_) cells, which presumably participate to disease development (11). We also reported altered DC–T cell interaction, which resulted in a decreased production of interleukin (IL)-10 and heightened production of IL-17 by regulatory CD4^+^ T cells (Treg) (12). Altered conventional type 2 DC (cDC2) functions from B27-rat were notably characterized by a defect in interferon (IFN) signaling and decreased IL-10 and IL-27 production, all of which could contribute to biased T cell stimulation and differentiation (13).

IL-27 is a heterodimeric member of the IL-12/IL-23 family of cytokines composed of two subunits: IL-27p28 (also known as IL-27a or IL-30) and Epstein-Barr virus induced gene 3 (EBI3 also known as IL-27b) (14). It is mainly produced by activated antigen presenting cells (APCs), including DCs, and signals through IL-27R receptor also composed of two subunits: WSX-1 (alternatively named IL-27Rα or TCCR) and gp130 (IL-6Rβ). IL-27R is expressed by numerous cell populations such as T and B cells, DCs, monocytes and endothelial cells (15, 16). IL-27 binding to its receptor induces activation of the Janus kinase (JAK)/signal transducer and activator of transcription (STAT) and p38 mitogen-activated protein kinase (MAPK) pathways (17).

Early studies showed that IL-27 may act as a pro-inflammatory cytokine by inducing Th_1_ responses (14). Hence, IL-27 increased T-bet expression, a Th_1_-specific transcription factor, and induced IFN-γ production by naïve T cells (18). However subsequent studies revealed a more complex role of IL-27 in the regulation of T cell responses. Hence, it was shown that IL-27 could inhibit the differentiation of Th_17_ cells by antagonizing the expression of the Th_17_ master transcription factor, retinoic acid receptor-related orphan receptor gamma t (RORγt) (19, 20). IL-27 was also able to induce the differentiation of type 1 Treg (Tr_1_) producing IL-10 and exerting immunosuppressive properties on Th_17_ cells (21, 22).

Thus, we speculated that a reduced production of IL-27 by DCs from B27-rat could be a key element in the emergence of Th_17_-driven inflammatory disease in this model. Hence, several factors induced by IL-27 in Tregs and directly involved in IL-10 production by those cells, such as c-Maf, aryl hydrocarbon receptor and IL-21, were down-regulated in Treg from B27-rat, consistent with such interpretation (12). Noteworthy, polymorphisms of the *IL27A* gene coding for the p28 subunit of IL-27 have been associated with SpA predisposition (23). In this study our objective was to investigate the effect of IL-27 on SpA, using the B27-rat model, to determine if this cytokine would be a suitable therapy target for SpA.

## Materials and methods

### Rats

The 33-3 B27-rats bearing 55 copies of HLA-B*2705 and 28 copies of hβ_2_m on a Fisher (F344) background were bred and maintained under conventional conditions, as previously described. Nontransgenic (NTG) F344 littermates were used as controls. Inducible costimulatory molecule (ICOS) knockout (ICOS^-/-^) Sprague-Dawley (SD) rats were produced by Dr. Ignacio Anegon, using the Talen technology (24) ICOS^-/-^ B27-rats were obtained by back-crossing ICOS^-/-^ SD rats onto 33-3 rats for four generations. Age-and sex-matched rats (1-8 months) were used in each experiment.

### Rat cells preparation

Single-cell suspensions were prepared from mesenteric or popliteal lymph nodes (LN), followed by flow cytometric analysis using a FACSAria cell sorter. Appropriate combinations of antibodies were used to identify Treg (CD4^+^ CD25^high^), resting T effector (Teff) cells (CD4^+^ CD25^-^ CD62L^-^), activated Teff cells (CD4^+^ CD25^low^ CD62L^-^), activated T cells (CD4^+^ CD25^+^) and naïve T cells (CD4^+^ CD25^-^ CD62L^+^) as shown in **Supplementary Figure S1**. Forkhead box P3 (Foxp3) staining using Alexa700 anti-mouse Foxp3 was performed according to manufacturer’s instructions (eBioscience) and the proportion of Foxp3^+^ Treg among CD4^+^ CD25^high^ T cells was assessed by flow cytometry, showing that the majority (>90%) of them were Foxp3^+^ (**Supplementary Figure S2B**). CD103^+^ cDCs were obtained from spleen as previously described (25, 26). Briefly, spleens were digested with Collagenase D (2mg/mL; Roche Diagnostics). After centrifugation on a 14.5% Nycodenz gradient (Nycomed), positive selection was performed by magnetic cell sorting with anti-rat CD103 microbeads with an AutoMacs Pro Separator (Miltenyi Biotec). The cDC2 were sorted from the purified CD103^+^ DCs based on CD4^+^ staining, on a FACSAria III sorter (BD Biosciences).

### Culture of rat CD4^+^ T cells

Flow cytometry-sorted activated CD4^+^ CD25^+^ T cells or resting CD4^+^ CD25^-^ CD62L^-^ Teff cells were cultured (10^5^ cells/well) in anti-CD3-coated (2 µg/mL) plates in the presence or not of anti-CD28 (1µg/mL) and rmIL-27 (5ng/mL). In some experiments, flow cytometry-sorted activated CD4^+^ CD25^+^ T cells were stimulated with coated-anti-CD3 (2 µg/mL) in the presence of JAK inhibitor (tofacitinib -anti-JAK1/3- 60 mM/mL or AG490 -anti-JAK2- 10 µg/mL). After 3 days, supernatants were collected to analyze cytokines levels by enzyme-linked immunosorbent assays (ELISA) and cells were restimulated for 4 hours with PMA (10 ng/mL), ionomycin (1 µg/mL) in the pressence of BFA (2 µg/mL) (Sigma) for intracellular cytokine and RORγt staining assay performed according to manufacturer’s instructions (BD pharmingen) (**Supplementary Figure S2A**). Stained cells were analyzed with a BD LSR Fortessa cell analyzer (BD Biosciences) using FlowJo software (TreeStar). All reagents and antibodies used in cell cultures are listed in **Supplementary Table SI**.

### Coculture of rat CD4^+^ T cells with DCs

Flow cytometry-sorted CD4^+^ CD25^high^ Treg or resting CD4^+^ CD25^-^ CD62L^-^ Teff cells were cultured (10^5^ cells/well) with sorted CD103^+^splenic DCs (0.4 x 10^5^ cells/well) pre-treated with mitomycin C (0.5 mg/mL) in anti-CD3-coated plates (2 µg/mL) in the presence or not of mrIL-27 (5ng/mL). After 3 days, supernatants were collected to analyze cytokine levels by ELISA and cells were restimulated for 4 hours with PMA (10 ng/mL), ionomycin (1 µg/mL) in the presence of BFA (2 µg/mL) for intracellular cytokine staining assay (**Supplementary Figure S3**). Stained cells were analyzed as above. For RNAseq analysis, flow cytometry-sorted CD4^+^ CD25^-^ CD62L^+^ naive T cells were cultured (10^5^ cells/well) with sorted splenic cDC2s (CD103^+^ CD4^+^; 10^4^ cells/well) and stimulated with coated-anti-CD3 (2 µg/mL) in the presence or not of rmIL-27 (5 ng/mL), AG490 (10 µg/mL) or tofacitinib (60 mM/mL). After 24 h of culture T cells were sorted by flow cytometry for RNA extraction.

### Rat CD4^+^ T cell differentiation assays

For Treg differentiation experiments, flow cytometry-sorted naïve CD4^+^ T cells (CD25^-^ CD62L^+^) were cultured (5 x 10^5^ cells/well) with CD103^+^ splenic DCs (10^5^ cells/well) pre-treated with mitomycin C at 0.5 mg/ml, in the presence of coated-anti-CD3 (2 µg/ml), rhTGF-β (2 ng/ml) and neutralizing anti-IFN-γ and anti-IL-4 antibodies (each at 10 µg/mL). For Th_17_ differentiation experiments, rrIL-6, rrIL-1β and rrIL-23 (each at 20 ng/mL) were added to the coculture. In some experiments, rmIL-27 (5 ng/mL) was also added to the coculture. At day 3, the medium was replaced by fresh medium supplemented with rrIL-2 (20 ng/mL). At day 6, supernatants were harvested for analysis of cytokine production by ELISA and cells were stimulated with PMA/ionomycin/BFA for intracellular cytokine and RORγt staining. In some experiments, 6 days after Th_17_ differentiation, T cells were sorted by flow cytometry for RNA extraction and RNAseq analysis.

### Human cells preparation and culture

Peripheral blood mononuclear cells (PBMC) were isolated from healthy controls and HLA-B27^+^ SpA patients (all fulfilling Assessment of SpondyloArthritis international Society classification criteria for axial SpA (27)) by Ficoll (GE Healthcare) density gradient centrifugation. The characteristics of patients and controls (unmatched for age) are shown in **Supplementary Table SII**). Patients were considered as resistant to conventional treatment if they had a Bath Ankylosing Spondylitis disease activity score > 3/10 despite non-steroidal anti-inflammatory drug and biotherapy usage. CD14^+^ cells were first removed from PBMC by positive magnetic selection, using anti-human CD14 microbeads and AutoMacs Pro Separator (Miltenyi). Then, CD4^+^ T cells were magnetically sorted from the CD14^-^ fraction using anti-human CD4 microbeads and AutoMacs Pro Separator (Miltenyi) and cultured during 6 days with rhIL-2 (10 ng/mL), soluble anti-CD28 (2 µg/mL) and coated-anti-CD3 (10 µg/mL) in the presence or absence of rhIL-27 (40 ng/mL) in complete medium. At day 6, supernatants were harvested for measure of cytokine levels by ELISA and cells were stimulated with PMA (10ng/mL), ionomycin (1µg/mL) and BFA (2µg/mL) for 6 hours for intracellular cytokine staining.

### Analysis of STAT1 and 3 phosphorylation by flow cytometry

Single-cell suspensions of rat mesenteric LN (10^6^ cells/tube) were stimulated with rmIL-27 (5 ng/mL) or PBS for 10 minutes at 37°C. After fixation during 10 minutes with Fix I Buffer (BD Biosciences) and permeabilization with Perm III buffer (BD Biosciences) for 30 minutes, cells were washed and stained with BD PhosFlow antibodies to pSTAT1 (pY701) and pSTAT3 (pY705) according to the manufacturer’s instructions (BD Biosciences)

### Determination of STAT1 and 3 intra-cellular content by flow cytometry

Total STAT content was determined with anti-STAT1 and anti-STAT3 (BD Biosciences) according to manufacturer’s instructions. Briefly, cells were stained using appropriate combinations of antibodies to identify Treg, Teff, and activated CD4^+^ T cells as described above. After fixation in Fix I Buffer (BD Biosciences) for 10 min and permeabilization with Perm III buffer (BD Biosciences) for 30 min, cells were washed and incubated with anti-STAT1 and STAT3 antibodies or isotypes control (**Supplementary Figure S4**) for 1h. After extensive washing, the fluorescence was quantified by BD LSR Fortessa cell analyzer.

### Determination of cytokine levels by ELISA

Rat and human IL-17, IL-10 and IFN-γ levels were determined in culture supernatants or serum by sandwich ELISA (IL-17: eBioscience; IL-10 and IFN-γ: R&D Systems) according to the manufacturer’s instructions. Concentrations were calculated according to calibration curves established from serial dilutions of recombinant standards in each assay. The sensitivity threshold of each cytokine assay was 10 pg/mL. Multiplex ELISA assay was performed using Bio-Plex Pro Rat Cytokine Panel (Biorad) according to the manufacturer’s instructions.

### Rat IL-27 recombinant production

The rrIL27 was prepared using a pTT3-IL-27 construction with 6 histidine signal peptide sequence. For the production the rrIL27, CHO cells were cultured in « FreeStyle™ CHO Expression Medium » (Life Technologies). Cells were thawed and subcultured three times prior to transfection. The transfection was performed using 1 μg of DNA per mL of cells culture. A volume of 2L was transfected. Cell density and viability were monitored regularly using Vi-CELLTM XR automatic cell counter and samples of supernatant collected and stored at 4°C for expression analysis by Western blot in denaturing and reducing conditions on precast Bis-Tris SDS-PAGE 4-12% (Life Technologies). Once the viability had dropped below 75% or 8 days after transfection, cell cultures were stopped. The supernatant was collected by centrifugation, filtered on 0.2 μm filter (Pall; PES) and loaded on 5 mL Ni Sepharose Excel prepacked column (GE Healthcare). The protein was eluted in step with PBS + 250 mM imidazole. The pool obtained from Ni Sepharose was dialyzed against sodium phosphate-NaCl buffer. The total protein concentration was determined using Bradford protocol with bovine serum albumin as a standard BCA assay (Pierce) and IL-27-specific ELISA assay. To produce sufficient quantities of rrIL-27 for the preclinical assay, we requested the service of a specialized company (PX-therapeutics).

### Preclinical assay

Sex-matched B27-rats and NTG F344 littermates (6-8 animals/group) aged of 4-6 weeks were randomized three days before initiation of the treatment, with a permutation table (each cage contained at least one rat from each experimental group). RrIL-27 (5 µg/rat/injection) or vehicle (PBS) were administered intraperitoneally 3 days per week for 10 consecutive weeks (experiment 1) or every day for 12 consecutive weeks (experiment 2). The rats were examined three times per week for clinical symptoms of colitis (loose stools and/or frank diarrhea) and arthritis and were assigned for each of these symptoms a score on a scale ranging from 0 (normal) to 4, as previously described (28). After 10 or 12 weeks of treatment, the rats were sacrificed. Colonic tissues were fixed in 4% phosphate-buffered formalin embedded in paraffin. Sections (4µm) were prepared and stained with hematoxylin-eosin (H&E). Tissues damages were semi-quantitatively scored blindly, following microscopic scoring criteria as previously described (28). The hindpaws of rats were excised and fixed in 4% phosphate-buffered formalin. After 24 h of fixation they were immersed for 3 weeks in Decal solution (Serva). After decalcification they were sectioned and stained with H&E. Sections were semi-quantitatively graded based on the degree of inflammation and joint destruction. Histologic findings were evaluated using modified grading scales, as previously described (29). Briefly, a 0–3 scale was used for gradation (where 0 is normal and 3 severe involvement) of synovitis and cartilage or bone structural modifications. Both ankle and tarsus were analyzed. The intracytoplasmic cytokines production was determined by flow cytometry in CD4^+^ T cells after PMA/ionomycin/BFA treatment

### RNA preparation, sequencing, and RNA-seq differential expression analysis

RNA was extracted from 10^5^ – 2 x 10^5^ Th_17_-polarizing or naïve CD4^+^ T cells cocultured with cDC2s, using the RNeasy Plus Micro Kit (Qiagen). RNA concentrations were obtained using a fluorometric Qubit RNA assay (Life Technologies). The quality of the RNA integrity number (RIN) was determined on the Agilent 2100 Bioanalyzer (Agilent Technologies, Palo Alto, CA, USA) as per the manufacturer’s instructions. To construct the libraries, 1 µg of high-quality total RNA sample (RIN > 8) was processed using TruSeq Stranded mRNA kit (Illumina) according to manufacturer instructions. Library Quantification Kit for Illumina Libraries (KapaBiosystems) and library profiles were assessed using the DNA High Sensitivity LabChip kit on an Agilent Bioanalyzer. Libraries were sequenced on an Illumina Nextseq 500 instrument using 75 base-lengths read V2 chemistry in a paired-end mode. After sequencing, a primary analysis based on AOZAN software (Ecole Normale Supérieure, Paris) was applied to demultiplex and control the quality of the raw data (based of FastQC modules/version 0.11.5). Obtained fastq files were then aligned using STAR (30) algorithm (version 2.5.2b). Reads were then counted using RSEM (31) (v1.2.28) and the statistical analyses on the read counts were performed with the R package DESeq2 (32) (v1.22.1) to determine the proportion of differentially expressed genes between two conditions. Differentially expressed genes identified by DESeq2 were submitted to Ingenuity Pathway Analysis (IPA) for pathway analysis using the Ingenuity Knowledge Base (Qiagen Bioinformatics)

### Statistical analysis

Data are expressed as the mean ± SEM. Statistical comparisons between groups were determined by a two-tailed paired Student’s *t* test, the Mann-Whitney or Wilcoxon nonparametric U test. *P* values < 0.05 were considered significant

### Study approval

Study procedures in rats were approved by the institutional animal experimentation ethical committee (APAFIS-8910) and Written informed consent was obtained from all subjects before study which was approved by the local ethical committee (AOR10006-NI09031).

## Results

### IL-27 reverses imbalanced production of IL-17 and IL-10 by CD4^+^ T cell subsets from B27 rats

**Figure 1:**
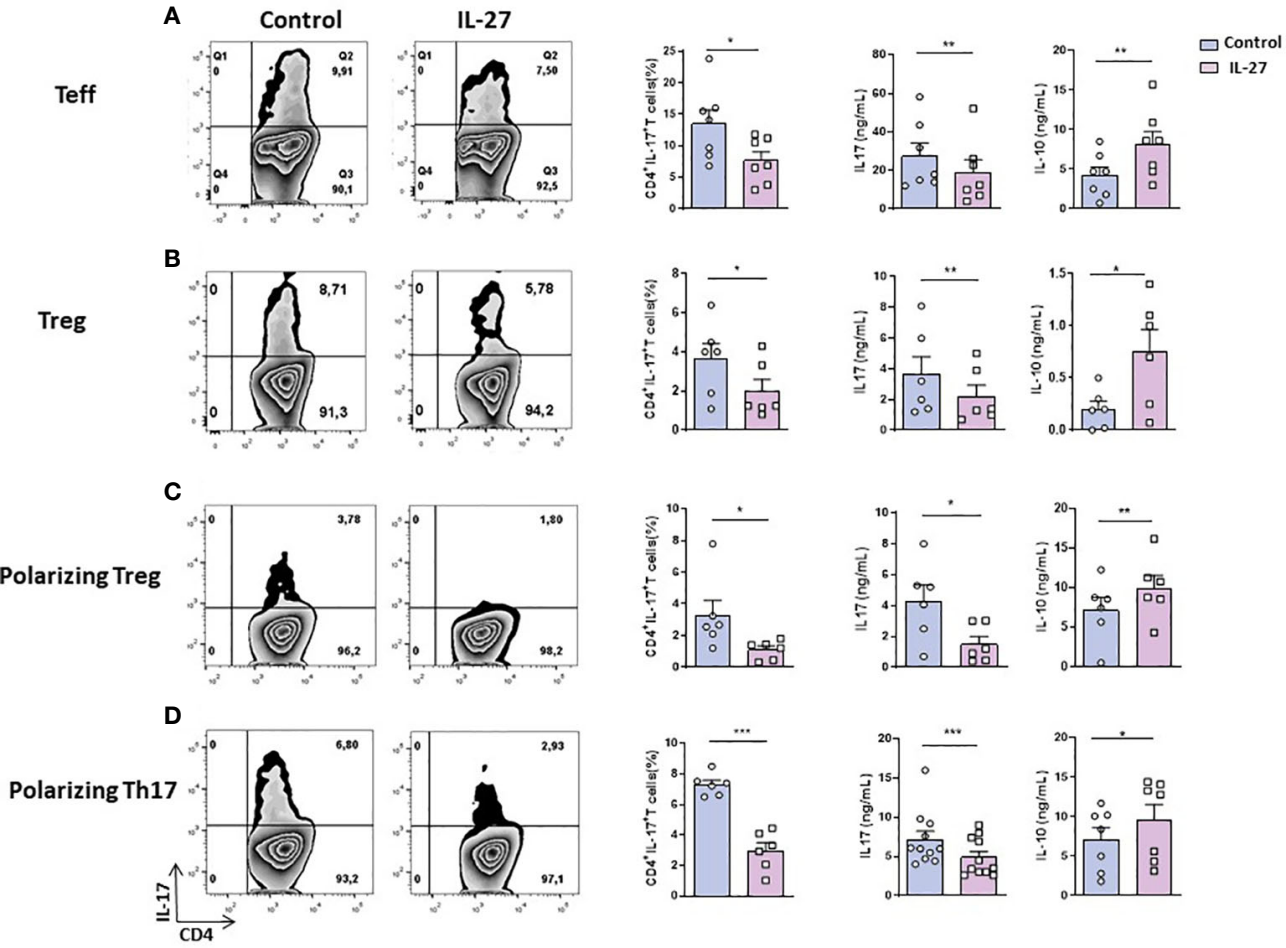
IL-27 effect on the generation/maintenance of IL-17-producing T cells. **(A)** *Ex vivo* sorted resting Teff cells (CD4^+^ CD25^-^ CD62L^-^ or **(B)** *ex vivo* sorted Treg (CD4^+^ CD25^high^) from B27-rats aged of 3-5 months were cocultured with CD103^+^ DCs for 3 days; **(C, D)** *ex vivo* sorted naïve (CD4^+^ CD25^-^ CD62L^+^) T cells were cocultured with CD103^+^ DCs for 6 days in **(C)** Treg (TGF-β) or **(D)** Th_17_ (TGF-β + IL-6 + IL-1β + IL-23) polarizing conditions. Left panels show representative flow cytometry dot plots evaluating the percentage of IL-17-producing CD4^+^ T cells after coculture and the mean + SEM of 6-11 rats/group; right panels show the mean + SEM of IL-17 and IL-10 levels that were evaluated in the supernatant of cocultures by ELISA. *p < 0.05; **p < 0.01 ; ***p = 0.0001..

Given that CD4+ T cells are induced to produce heightened levels of IL-17 (9) in contact with cDCs and that cDCs exhibit decreased production of IL-27 (13) in B27-rat, we sought to determine whether exogenous IL-27 could exert antagonistic effect on such T cell bias. Indeed, coculture of resting Teff cells (CD4^+^ CD25^-^ CD62L^-^) with CD103^+^ DCs from B27-rat in the presence of rmIL-27 resulted in reduced IL-17 production (**Figure 1A**). Increased IL-10 level was also detected in supernatants of those cocultures (**Figure 1A**). To investigate next the effect of IL-27 on imbalanced production of IL-17 and IL-10 by Treg from B27-rat (12), we performed cocultures of Treg (CD4^+^ CD25^high^) with CD103^+^ DCs from B27-rat. Hence, IL-27 addition decreased the frequency of IL-17 producing Treg and IL-17 levels produced in supernatant (**Figure 1B**). In parallel, we observed a significant increase of IL-10 production by Treg from B27-rats in the presence of IL-27 (**Figure 1B**). Analysis by multiplex ELISA assay revealed that IL-17 and IL-10 were the only cytokines significantly modulated by IL-27 among a panel of 11 cytokines of interest (**Supplementary Figure S5**).

### IL-27 decreases IL-17^+^ T cells generation from B27-rat naïve CD4^+^ T cells

It was previously described that IL-27 can influence T cell differentiation by supporting Th_1_ and Tr_1_ differentiation and inhibiting Th_17_ commitment (16). Given that cDCs from B27-rat favor IL-17 production by Treg (12) and Th_17_ expansion (11) we tested if IL-27 could also inhibit *in vitro* differentiation of IL-17 producing-Treg from naïve CD4^+^ T cells. A decreased proportion of IL-17^+^ T cells was observed in the presence of rmIL-27, after coculture of naïve CD4^+^ T cells with CD103^+^ DCs under Treg polarizing condition (**Figure 1C**). Furthermore, a decreased production of IL-17 and an increased production of IL-10 were observed in coculture supernatants in the presence of IL-27 (**Figure 1C**). We also observed decreased frequency of RORγt^+^ T cells and increased frequency of IFN-γ-producing T cells and higher levels of IFN-γ in the supernatant (**Supplementary Figure S6B**), in the presence of IL-27. This confirmed an inhibitory effect of IL-27 on Th_17_ differentiation of naïve CD4^+^ T cells and conversely, a pro-Th_1_ role. Similarly, when naïve CD4^+^ T cells were cocultured with CD103^+^ DCs under Th_17_-polarizing conditions, addition of rmIL-27 reduced the proportion of IL-17 producing T cells and IL-17 production in supernatant, increasing significantly IL-10 levels (**Figure 1D**). In the latter conditions IL-27 also reduced the frequency of RORγt^+^ T cells (**Supplementary Figure S6C**) and increased IFN-γ production in culture supernatants (**Supplementary Figure S6D**).

Given that both cDC and CD4^+^ T cells express a functional IL-27R, we next examined which cell population(s) mediated IL-27 effects. Interestingly, we observed a direct inhibitory effect of IL-27 addition on the frequency of RORγt^+^ cells and on the production of IL-17 by effector T cells isolated from mesenteric LN of B27-rats, whether in an activated CD4^+^ CD25^low^ (**Supplementary Figure S7A**) or resting CD4^+^ CD25^-^ CD62L^-^ (**Supplementary Figure S7B**) state, in the absence of DCs. Alike the foregoing results observed for cocultures in the presence of cDCs, IL-10 production was also increased in the presence of IL-27 in those conditions, whereas IFN-γ production was decreased (**Supplementary Figure S7A, B**). Activated T cells from NTG rats produce little IL-17, as compared to those from B27-rats, however addition of IL-27 to cultures of those cells led also to a significant decrease of this cytokine in culture supernatants (data not shown). Conversely, IL-27 treatment of DCs before coculture had no effect on their expression of co-stimulatory (CD40, CD86) or MHC (class I and II) molecules nor on the capacity of those cells to expand IL-17^+^ T cells (data not shown).

### Anti-IL-17 effect of IL-27 on B27-rat CD4^+^ T cells is independent of IL-10 or ICOS

**Figure 2:**
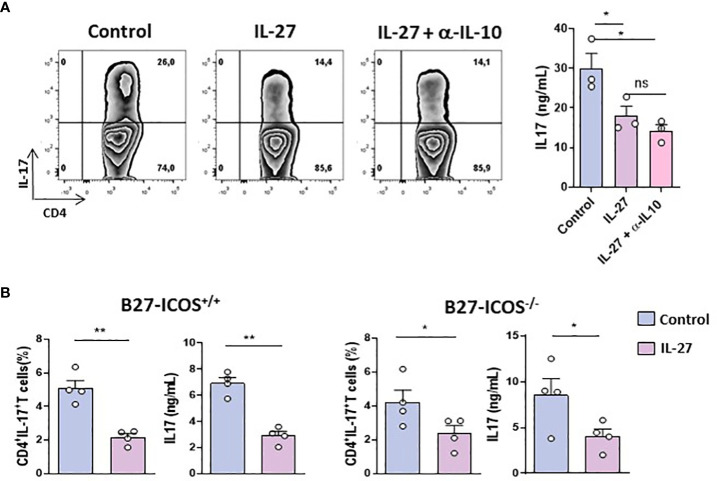
IL-27 inhibitory effect on B27-T cells is IL-10/ICOS independent. **(A)** Left: Representative dot-plot of IL-17^+^ cells among activated CD4^+^ CD25^+^ T cells from B27-rat aged of 5 months cultured for 3 days with coated anti-CD3 in the presence or absence of IL-27 and anti-IL-10 blocking antibody. Right panel: level of IL-17 in culture supernatants (3 rats/group). **(B)** CD4^+^ CD25^+^ T cells from ICOS^+/+^ or ICOS^-/-^ B27- rats (aged of 9-11 months) cultured for 3 days with coated anti-CD3 in the presence or not of rmIL-27. Histograms represent the mean + SEM of 3-4 rats/group. IL-17 and IL-10 concentrations were evaluated by ELISA. *p < 0.05; **p < 0.01. ns, not significant.

Given that IL-27 may induce the differentiation of IL-10-producing Tr_1_ and inhibit Th_17_ cells generation and that increased IL-10 production preceded inhibition of IL-17 in the foregoing *in vitro* studies (**Supplementary Figure S8A**), decreased IL-17 production by CD4^+^ T cells from B27-rat could be due to a negative feedback loop induced by IL-10. To test this hypothesis, we studied IL-27 effect on activated CD4^+^ CD25^+^ T cells from B27-rat (because these cells produce quickly high levels of IL-17 after stimulation) cultured in the presence of IL-10-neutralizing antibody. The presence of anti-IL-10 antibody did not prevent the inhibitory effect of IL-27 on IL-17 production (**Figure 2A**). Moreover, IL-27 inhibited the generation of IL-17^+^ T cells from naïve CD4^+^ T cells under Th_17_ polarizing conditions to a similar extent when IL-10 was blocked (**Supplementary Figure S8B**). Hence, the inhibitory effect of IL-27 on IL-17 production and Th_17_ cells differentiation is IL-10-independent. We previously showed that blockade of ICOS/ICOSL pathway resulted in increased IL-10 and decreased IL-17 productions by CD4^+^ T cells from B27-rats (12). Interestingly, in the presence of IL-27, we observed *in vitro* a decreased expression of ICOS on activated CD4^+^CD25^+^ T cells and on naïve CD4^+^ T cells cultured in Treg or Th_17_ polarizing conditions (**Supplementary Figure S8C**). Therefore, we cultured sorted CD4^+^ CD25^+^ T cells from B27-rats bearing ICOS deletion (ICOS^-/-^) in the presence of IL-27, to test if IL-27 effect could be secondary to ICOS down-regulation. Addition of IL-27 reduced the proportion of IL-17^+^ cells and IL-17 production after 3 days of culture, in CD4^+^ CD25^+^ cells from both ICOS^+/+^ and ICOS^-/-^ B27-rats to similar extent (**Figure 2B**). Thus, the absence of ICOS on CD4^+^ T cells from B27-rat did not influence the effect of IL-27 on those cells.

### IL-27 induces similar STAT1/3 phosphorylation in NTG and B27-rat CD4^+^ T cells despite increased levels of STAT3 in B27-rat

**Figure 3:**
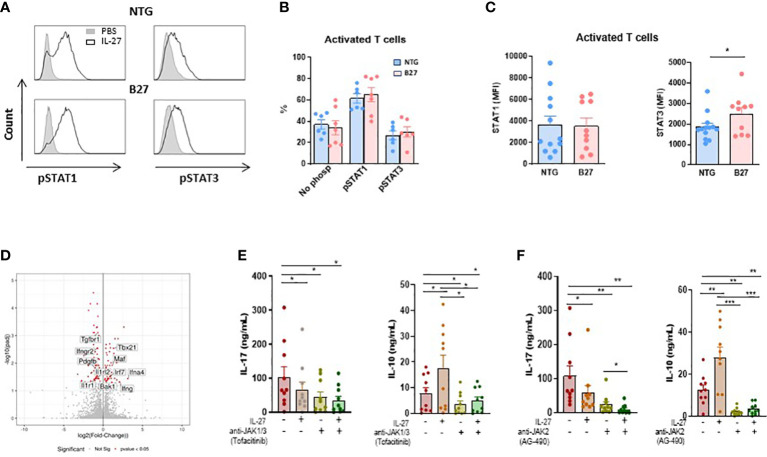
JAK-STAT signaling is involved in IL-27 modulatory effect. **(A)** Representative histogram of mesenteric LN cells stimulated by IL-27 and stained intracellularly for phosphorylated STAT1 and STAT3 gated on CD4^+^ CD25^+^ activated T cells. **(B)** The percentage of pSTAT1 and pSTAT3 was analyzed in gated CD4^+^ CD25^+^ activated T cells from mesenteric LN of NTG and B27-rat aged of 4-5 months. **(C)** STAT1 and STAT3 levels were analyzed by flow cytometry in gated CD4^+^ CD25^+^ activated T cells from mesenteric LN of NTG and B27 rat. Values are the mean + SEM of 6-13 rats/group. *p<0.05. **(D)** Volcano plot representing the results of RNAseq analysis of differentiated Th_17_ cells in the presence of IL-27, as compared to medium control. The data for all genes are represented by log2 (fold change) *vs.* log10 of adjusted p-value. The significative different genes are highlighted in red. Values represent results from 5 rats/group. **(E, F)** IL-17 and IL-10 levels were determined by ELISA in supernatants from CD4^+^ CD25^+^ activated T cells after 3 days of stimulation with coated anti-CD3 in the presence of IL-27 and/or anti-JAK1/3-tofacitinib **(E)** and/or anti-JAK2-AG490 **(F)**. Values are the mean + SEM of 10 rats/group. *p < 0.05; **p = 0.001; ***p = 0.0005.

It has been shown that IL-27 induced the phosphorylation of STAT1 and STAT3 after binding to IL-27R and that activation of those STATs was required for inhibition of Th_17_ differentiation and induction of IL-10 production (22). To examine if such mechanism applied to IL-27 effect on B27-rat CD4^+^ T cells, we studied the phosphorylation of STAT1 and STAT3 by flow cytometry after IL-27 stimulation in several T cells subsets. IL-27 induced the phosphorylation of STAT1 and STAT3 in approximatively 60% and 30% of activated CD4^+^ T cells, respectively, without difference between NTG and B27-rat (**Figures 3A, B**). Similar results were observed with Treg and Teff cells (**Supplementary Figure S9A**). These results indicate that IL-27 stimulation has a similar capacity to induce pSTAT1/3 in CD4^+^ T cell subsets from NTG and B27-rats. Interestingly however, when we analyzed total STAT1/3 levels in CD4^+^ T cells we observed an increased level of STAT3 in activated T cells (**Figure 3C**), Treg and Teff cells (**Supplementary Figure S9B**) from B27-rats which may contribute to the increased IL-17 production.

### JAK-STAT signaling mediates IL-10 increase induced by IL-27 in activated CD4^+^ T cells from B27-rat

To define potential mechanisms by which IL-27 priming inhibited Th_17_ cell differentiation we performed RNA-sequencing (RNA-seq) in naïve CD4^+^ T cells cocultured with CD103^+^ DCs under Th_17_-polarizing condition in the presence or not of IL-27. We found that 109 genes were down-regulated and 68 were upregulated in Th_17_-differenciated cells in the presence of IL-27, as compared to control condition (**Figure 3D**). Interestingly, IL-27 negatively modulated several genes which have been described to be involved in the pathogenesis of SpA and/or the STAT3 pathway, a transcription factor which is increased in CD4^+^ T cells from B27-rat (**Figure 3C** and **Supplementary Figure S9A, B**) and plays a major role in the differentiation of Th_17_ cells, including *Il1r1, Ilrl2*, *Pdgfb* and *Tgfbr1* (33), (**Figure 3D**). On the other hand, we observed an increased expression of *Maf* gene (**Figure 3D**), which is downregulated in B27-rat Treg (12). Interestingly, this gene codes for c-maf, a positive regulator of IL-10 production and may thereby inhibit Th_17_ response (34). STAT3 dysfunction has been described in various chronic inflammatory diseases and JAK-STAT inhibitors are effective in treating several of these disorders, including SpA (35, 36). The foregoing RNAseq analysis suggested that the inhibitory effect of IL-27 on IL-17 production was associated with STAT3 pathway down-regulation. To test this hypothesis, we performed cultures of activated CD4^+^ T cells in the presence of IL-27 and/or JAK-STAT inhibitors. Given that IL-27 induces the dimerization of gp130 and IL-27Ra that engages JAK1 and JAK2, thereby activating both STAT1 and STAT3, we performed experiments with tofacitinib, JAK1/3-STAT1 and AG490, JAK2-STAT3 inhibitors, respectively. IL-17 production was reduced in the presence of IL-27 but also in the presence of both JAK-inhibitors alone (**Figures 3E, F**). Interestingly, we observed an additive effect between IL-27 and AG490, suggesting that factor(s) independent of STAT3 likely contributed to the decreased production of IL-17 induced by IL-27 (**Figure 3F**). In contrast, the ability of IL-27 to induce IL-10 appeared fully dependent on JAK-STAT activation because IL-10 production induced by IL-27 was abolished in the presence of JAK-STAT inhibitors (**Figures 3E, F**).

### IL-27 modulatory effect is associated with STAT1/STAT3 balance restoration

**Figure 4:**
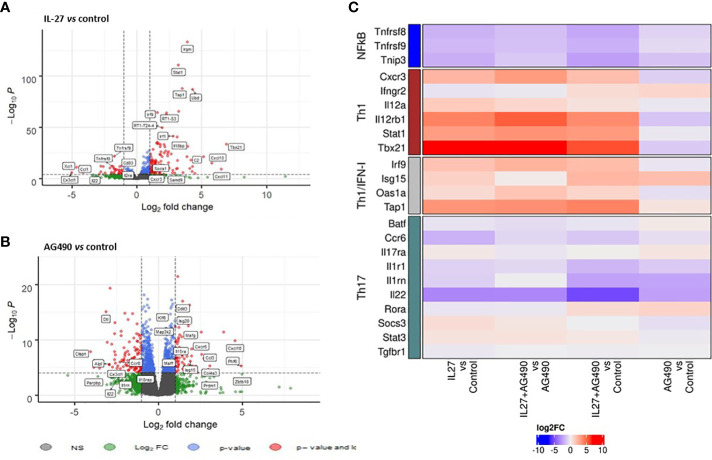
IL-27 modulatory effect is associated with STAT1/STAT3 balance restoration. Naïve T cells that had been coculture with splenic cDC2s and coated-anti-CD3, in the presence or not of mrIL-27 (5 ng/mL), AG490 (10 µg/mL) or both for 24 h were sorted. RNA was isolated and global gene expression was determined by RNA-seq. **(A)** volcano plot represents IL-27 condition compared to medium control and **(B)** AG490 condition compared to medium control. **(A, B)** The data for all genes are represented by log2 (fold change) *vs* log10 of adjusted p-value. **(C)** A heatmap of fold change (log2) for genes differentially expressed between conditions is shown. Values represent results from of 5 rats aged of 4-5 months per group.

Interestingly, we observed by RNAseq analysis of naïve CD4^+^ T cells cocultures that cDC2 from B27-rat promoted an inflammatory bias, as compared to NTG rat cDC2, independently of the addition of polarizing cytokines (i.e. TGF-β, IL-6, IL-1 and IL-23) (**Supplementary Figure. S10A**). This coculture system allowed us to study the modulatory effect of both IL-27 and AG490 on naïve CD4^+^ T cells developing without the possible confounding effect of exogenous cytokines addition. We observed that IL-27 downregulated some genes involved in Th_17_ response or nuclear factor-kappa B (NFκB) pathway, such as *Il22, Tnfrsf8*, *Tnfrsf9, Ccr6, Il1r1*. Conversely, IL-27 upregulated several genes known to promote Th_1_ response, including *Stat1, Tbx21, Irf1, Irf9, Cxcr3, Cxcl10*, *Cxcl11* and of *Socs1*, a negative regulator of cytokines signaling. These data indicate that IL-27 modulated the proinflammatory program instructed by B27-rat cDC2 to naïve CD4^+^ T cells by downregulation of genes associated to STAT3 pathway and upregulation of genes associated to Th_1_ responses (**Figures 4A, C** and **Supplementary Figure S10B**). The presence of AG490 in cocultures also negatively modulated *Il22* gene implicated in Th_17_ response and *Il1rn and Il18rap* (37) genes (**Figure 4B**). We also observed a downregulation of NFkB-associated genes only when AG490 was combined with IL-27. In this condition, the presence of IL-27 favored an upregulation of Th_1_-associated genes (*Stat1*, *Tbx21*, *Irf1* and *Irf9*), not observed in cells cultured only in the presence of AG490 (**Figure 4C**).

### IL-27 decreases IL-17 production by CD4^+^ T cells from SpA patients

**Figure 5:**
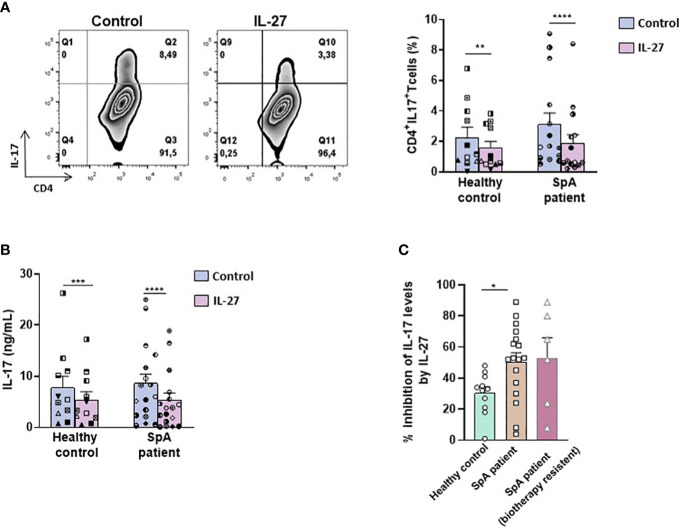
IL-27 decreases IL-17 production by T cells from SpA patients. **(A)** Left panel: representative dot-plot and right panel: percentage of IL-17 producing-CD4^+^ T cells from SpA patients cultured during 6 days with IL-2 in the presence or absence of IL-27 and determined after PMA/ionomycin stimulation. **(B)** IL-17 production was measured in culture supernatants by ELISA. **(C)** Percentage of inhibition of IL-17 production induced by IL-27 presence in cultures of CD4+T cells from healthy controls and SpA patients. *p < 0.05 **p<0.005 ****p<0.0001/ ***p=0.001.

Given that IL-27 reversed heightened production of IL-17 by several CD4^+^ T cell subsets in the B27-rat and that increased production of IL-17 appears to be characteristic of SpA patients (38), we wished to determine if IL-27 could exert similar effects on human T cells. Thus, we cultured purified CD4^+^ T cells isolated from the peripheral blood of SpA patients and healthy controls in the presence of rhIL-27 for 6 days. Similarly, to the results observed in B27-rat, the addition of rhIL-27 reduced the proportion of IL-17^+^ CD4^+^ human T cells in all 15 patients and 8/11 (73%) healthy controls (**Figure 5A**) and reduced the level of IL-17 in culture supernatant in 16/18 patients (89%) and 10/11 (91%) healthy controls (**Figure 5B**). Interestingly, the inhibitory effect of IL-27 was greater in SpA patients than healthy controls (**Figure 5C**), in such a way that the production of IL-17 was equivalent between both groups after exposure to IL-27 (**Figure 5B**). Remarkably, this anti-inflammatory effect of IL-27 was equally observed on CD4^+^ T cells from several SpA patients resistant to conventional treatment with non-steroidal anti-inflammatory drug and biotherapy. IL-27 reduced the percentage of IL-17^+^ T cells in all and the IL-17 levels in 5/6 treatment-resistant SpA patients (**Figure 5C**). However, in contrast to the results observed in B27-rat, no change of IL-10 or IFN-γ levels in supernatant was observed in the presence of IL-27 in cultures of SpA patients cells (**Supplementary Figure S11A, B**), suggesting that the effect of IL-27 on human CD4^+^ T cells display some differences compared to those observed in rat.

### RrIL-27 reduces SpA severity in B27-rat

**Figure 6:**
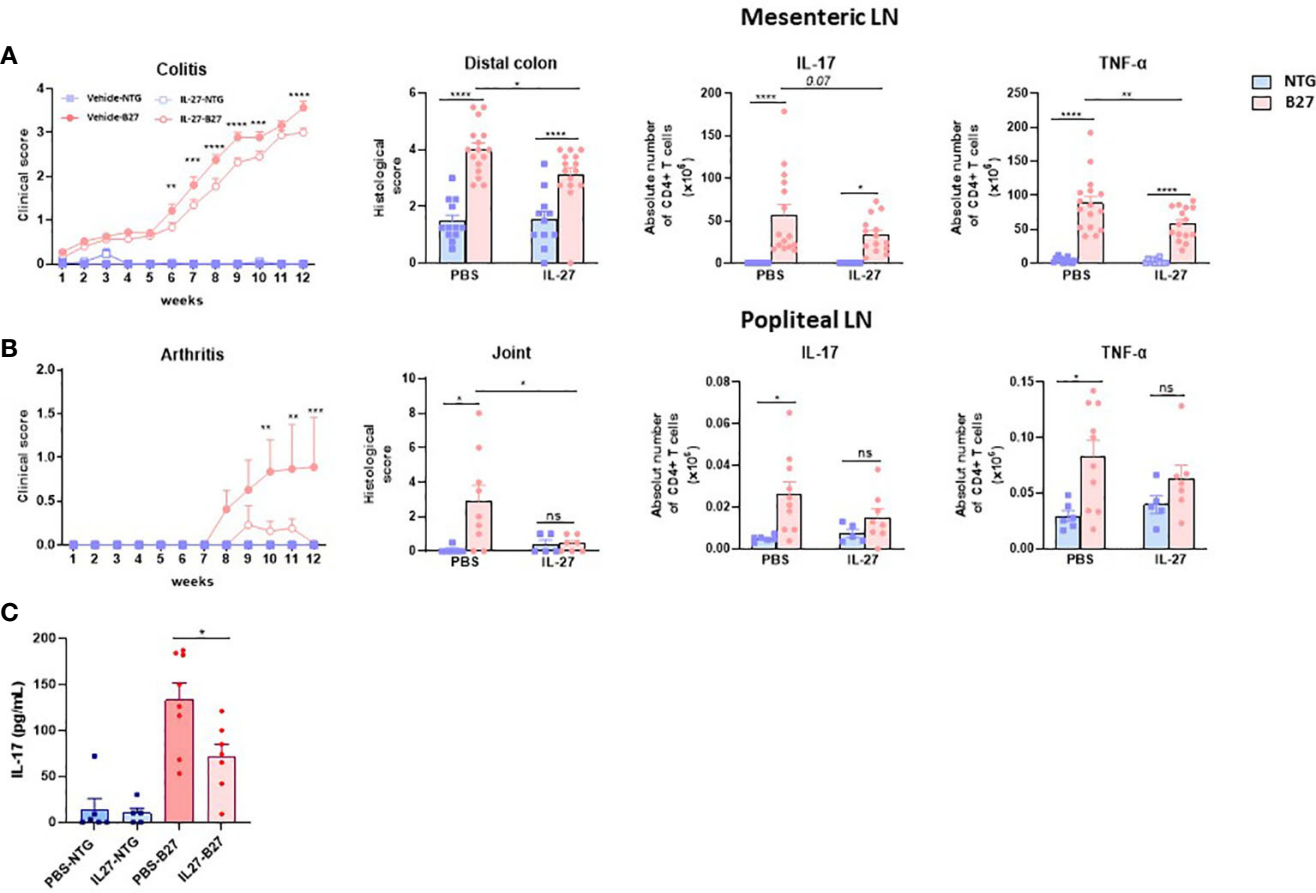
RrIL-27 reduces SpA severity in B27-rat. **(A)** Clinical score of colitis (experiment 2), histological score of distal colon inflammation and absolute number of cytokines producing T cells (pooled experiments 1 and 2) were determined in mesenteric LN from NTG and B27-rats aged of 4-6 weeks treated with vehicle or rrIL-27 for 12 weeks. **(B)** Clinical score of arthritis, histological score of hindpaws and the absolute number of cytokines producing T cells in popliteal LN were determined in NTG and B27-rats treated with vehicle or rrIL-27 for 12 weeks (experiment 2). **(C)** IL-17 levels in serum from NTG and B27 treated with PBS or IL-27. Values are the mean ± SEM of 6-8 rats/group. *p < 0.05 ***p<0.0005 ****p<0.0001. **p=0.002. ns, not significant.

To evaluate *in vivo* the effect of rrIL-27 on SpA development, we treated premorbid B27-rats and control NTG rats in two consecutive experiments with rrIL-27 or vehicle. In the first experiment, rats were treated three times per week for 10 weeks whereas they were treated daily for 12 weeks in the second experiment. NTG rats, whether treated or not with IL-27, remained healthy clinically and by histology, thereby indicating that IL-27 administration was well-tolerated. As expected, vehicle-treated B27-rats developed colitis symptoms characterized by chronic diarrhea that started at 6-7 weeks of age and progressively worsened over the treatment period. In both experiments, clinical score of colitis was reduced in the IL-27-treated B27-rats (**Figure 6A** and **Supplementary Figure S12A**). Histological examination of distal colon confirmed reduced inflammation in the IL-27-treated B27-rats (**Figure 6A** and **Supplementary Figure S12D**). In the first experiment, arthritis course was not significantly affected by IL-27 treatment (**Supplementary Figure S12B**), whereas the second more intense regimen of rrIL-27 administration was associated with inhibition of arthritis development (**Figure 6B** and **Supplementary Figure S12C**). Histological study of hindpaws exhibited significant inflammation and joint destruction only in the vehicle-treated B27-rats (**Figure 6B** and **Supplementary Figure S12E**). Interestingly, IL-27 treatment was associated with a striking reduction of the number of CD4^+^ T cells producing IL-17 or TNF-α in the mesenteric LN from B27-rats (**Figure 6A**). In the popliteal LN, the numbers of IL-17^+^ and TNF-α^+^ CD4^+^ T cells were significatively increased in the vehicle-treated but not in the IL-27-treated B27-rats, as compared to NTG controls. Furthermore, serum IL-17 levels were reduced in IL-27-treated, compared to control B27 rats (**Figure 6C**). Altogether, those data show a beneficial effect of IL-27 treatment on hallmark manifestations of SpA in the B27-rat model, most likely by reducing IL-17 and/or TNFα production.

## Discussion

Therapeutic options to treat SpA, a disabling chronic inflammatory disorder of the joint, are still limited despite recent advances that include the development of TNF-α, IL-17 and JAK inhibitory agents (36, 39). This is why B27-rat, considered as the most faithful animal model of SpA, is highly valuable in understanding the mechanisms of SpA and identifying new therapeutic targets (5, 40).This model was instrumental in highlighting CD4^+^ Th_17_ cells and heightened IL-17 production as major components of SpA pathophysiology (11, 36, 41). Moreover, B27-rat DCs exhibit aberrant features, including a decreased production of IFN-γ, IL-10 and IL-27, that could contribute to such deregulated CD4^+^ T cells phenotype (13). IL-10 treatment alone failed to improve rat SpA (28). Thus, we speculated that IL-27, a cytokine known to inhibit the differentiation of Th_17_ and induce the differentiation of IL-10 producing Tr_1_ could me a more suitable target (14).

Here, we first showed that IL-27 selectively opposed IL-17 and stimulated IL-10 production by several subsets of B27-rat CD4^+^ T cells, including *ex vivo* sorted resting and activated effector cells, Treg and *in vitro* polarized Th_17_ cells and Treg, and that this effect was independent of coculture with DCs. This effect on cytokines was associated with reduced frequency of T cells expressing RORγt^+^, the master transcriptional factor of Th_17_ cells, that is increased in B27-rat CD4^+^ T cells (11). Those results indicated that in addition to reduce IL-17 production by CD4^+^ Teff cells, IL-27 could reverse aberrant proinflammatory pattern of Treg by inhibiting excessive IL-17 and restoring IL-10 productions (12). Importantly, IL-27 also reduced the production of IL-17 by CD4^+^ T cells from SpA patients in culture, including those who were non-responders to currently approved biotherapies, indicating that IL-27 could be a promising therapy by opposing Th_17_ cells, even in case of conventional treatment failure.

Previous works demonstrated either a crucial role of IL-10 for the anti-inflammatory effect of IL-27 in a model of colitis or that IL-10 was not required for the suppression of Th_17_ cells in the context of experimental autoimmune encephalomyelitis (42, 43). Here, we found that IL-10 secretion was not mediating the modulatory effect of IL-27 on Th_17_ cells. It was also previously shown that the induction of Tr_1_ cells by IL-27 relied on the induction of three components: c-maf, IL-21 and ICOS (44). Here, IL-27 decreased ICOS expression on CD4^+^ T cells from B27 rats that exhibited increased ICOS expression, a feature that could participate to Th_17_ bias (12, 45). Nevertheless, using ICOS^-/-^ B27-rats, we observed that the modulatory effect of IL-27 was independent of ICOS.

To dissect further the mechanisms by which IL-27 may oppose Th_17_ differentiation bias favored by B27-rat DCs, we studied the effect of IL-27 on the transcriptome of naïve CD4^+^ T cells having been differentiated in contact with B27-rat DCs in Th_17_-polarizing conditions. Interestingly, IL-27 modulated negatively genes associated to STAT3 signaling that are likely to control the inflammatory response involved in the pathogenesis of SpA. Conversely, *Maf* and several *Ifng*-associated genes, including *Tbx21*, antagonizing Th_17_ response were up-regulated (34, 46). This is consistent with STAT1 phosphorylation induced by IL-27 and enhanced production of IFN-γ that was observed in this culture condition, albeit the inhibitory effect of IL-27 on Th_17_ pathway was not mediated by IFN-γ itself, given the presence of anti-IFN-γ in the culture. Noteworthy, in contrast to increased production in Treg or Th_17_ polarizing conditions, IFN-γ production was decreased by IL-27 in differentiated CD4^+^ T cells which is consistent with the capacity of IL-27 to limit excessive Th_1_ response by induction of IL-10 expression (47).

To examine whether JAK-STAT signaling transduced IL-27 effects in this model, we added inhibitors of JAK1/3 (tofacitinib, a molecule approved for SpA treatment) or JAK2 (AG490) that preferentially target STAT1 and STAT3, respectively. Both inhibitors reduced IL-17 and IL-10 production by activated CD4^+^ effector T cells from B27-rat, as a likely consequence of STAT3 inhibition. This precluded us to determine if IL-27 inhibited IL-17 production through STAT signaling. However, increased production of IL-10 by IL-27 was abolished in the presence of both STAT inhibitors, showing that it was dependent on STAT signaling. Moreover, there was an additive effect of IL-27 and JAK2 inhibition on IL-17 inhibition, which might reflect the combination of STAT1 phosphorylation by IL-27 and STAT3 inhibition by AG490. Altogether, those results indicate that IL-27 exerted more thorough anti-inflammatory effect on CD4^+^ T cells from B27-rat than JAK inhibitors, by combining IL-17 inhibition to IL-10 up-regulation.

We then analyzed the effect of IL-27 and/or AG490 in a simplified coculture system where cDC2 from B27-rat drives a Th_17_ bias in naïve CD4^+^ T cells without exogenously added cytokines, which may underly the pathophysiology of B27-rat SpA. Using again RNAseq, we interrogated the early transcriptional events occurring after 24h of naïve CD4^+^ T cell stimulation. This confirmed that IL-27 upregulated STAT1-IFN-pathway associated genes and downregulated genes associated to Th_17_ program, consistent with a mechanism involving a greater phosphorylation of STAT1 than STAT3 by IL-27 (48) (**Figure 3A**). This effect of IL-27 resulted in NFκB pathway inhibition. AG490 also inhibited Th_17_ pathway and NFκB but to a lesser degree than IL-27 and was rather inhibitory on genes of the STAT1-IFN pathway.

Given the striking *in vitro* anti-IL-17 and pro-IL-10 effect of IL-27 on CD4^+^ T cells from B27 rats, IL-27 appeared a promising therapeutic agent in SpA. Indeed, *in vivo* treatment of B27-rats with exogenous IL-27 was able to inhibit SpA development and the expansion of IL-17 and TNF-α producing Teff cells in a dose-dependent manner. Hence, a high dose of IL-27 administered daily could effectively inhibit both intestinal inflammation and arthritis, the two main pathologic features in this rat model of SpA. This result is consistent with a beneficial effect of IL-27 previously reported in others experimental models of arthritis or colitis involving Th_17_ cells (49, 50). However, it is all the more remarkable that neither administration of anti-IL-17 antibody (11), rmIL-10 (28) nor low dose of rhIL-2 to stimulate Treg (29) were sufficient to counter the development of rat SpA.

Conventional Th17 corresponding to CD4^+^T cells are a major source of IL-17. However, other cellular types may also produce this inflammatory cytokine, and most of them belong to the innate like-immune system. Recently, a number of specialized cells, such as innate-like T-cells, innate lymphoid cells (ILCs), mucosal-associated invariant T cell (MAIT) and natural killer receptor (NKR)- expressing T cells, have been marked to be involved in SpA pathology (51). Thus, it will be interesting to assess the effect of IL-27 on these cells in further studies.

In summary we demonstrated for the first time that exogenous IL-27 reversed the pro-Th_17_ phenotype characteristic of the B27-rat model of SpA. Such effect was demonstrated both on the developing and differentiated IL-17 producing CD4^+^ T cells *in vitro*. Moreover, similar modulatory effect was also observed in CD4^+^ T cells from SpA patients. Finally, *in vivo* administration of rrIL-27 inhibited the development of SpA in B27-rat, in which DCs exhibit deficient IL-27 production (13). Taken together, these results highlight a critical modulatory role of IL-27 on the emergence of Th_17_-driven inflammatory disease in B27 rats and offer a novel therapeutic perspective for SpA patients, including those who are refractory to currently available treatments.

## Data availability statement

The data presented in the study are deposited in the European Nucleotide Archive repository, accession number PRJEB56819.

## Ethics statement

Written informed consent was obtained from all subjects before study which was approved by the local ethical committee (AOR10006-NI09031). Study procedures in rats were approved by the institutional animal experimentation ethical committee (APAFIS-8910).

## Author contributions

QJ and LA designed research studies, conducted experiments, acquired data, analyzed data and wrote the manuscript, BC, AJ-M, GM, BH, and FL conducted experiments, acquired data and analyzed data. SG, ML, KE, and BI conducted experiments and acquired data, BS, analyzed data, SR and IA provided Icos KO rats, AE provided IL-27rr batch, M-CB, analyzed data, GC, designed research studies, MB designed research studies and wrote the manuscript. All authors contributed to the article and approved the submitted version.
