# Supplementary material for: Interleukin 27 is a novel cytokine with anti-inflammatory effects against spondyloarthritis through the suppression of Th17 responses
Source: Front Immunol. 2023 Feb 2;13:1072420. doi: 10.3389/fimmu.2022.1072420 (PMC9933703; doi:10.3389/fimmu.2022.1072420)
Supplement: Supplementary file 1 [file DataSheet_1.docx]

Gating strategy

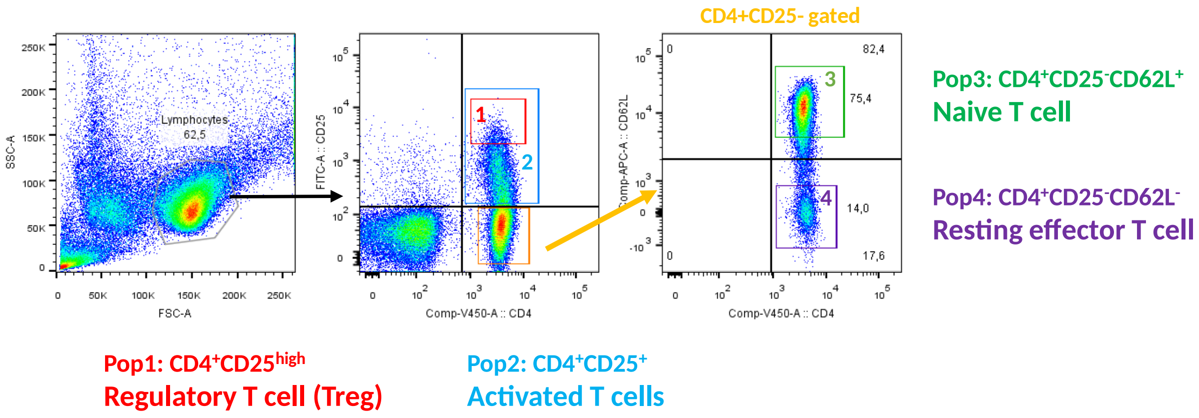

Supplementary Figure 1: Gating strategy: Flow cytometry was performed in lymph node cell suspension using a combination of anti-CD4, anti-CD25 and anti-CD62L to distinguish and/or sort 4 populations of $CD4^+$ T cells, namely, $CD4^+CD25^{High}$ Treg, $CD4^+CD25^+$ activated T cells, $CD4^+CD25^-CD62L^+$ naive T cells and $CD4^+CD25^-CD62L^-$ resting effector T cell.

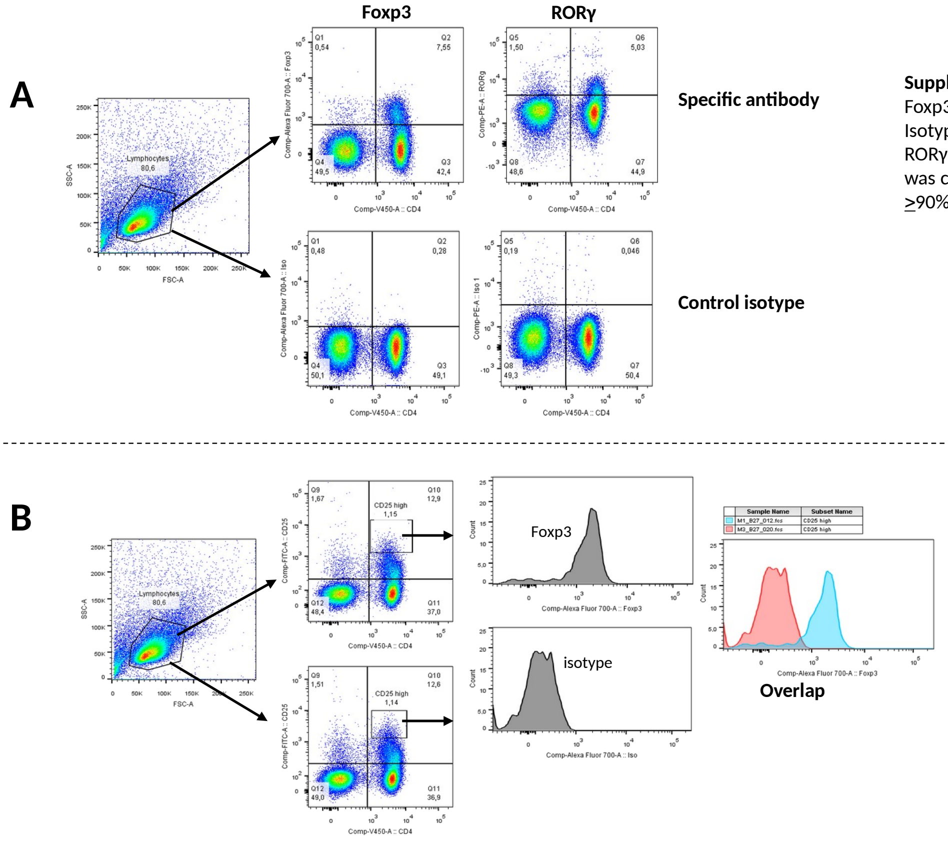

Supplementary Figure 2: A-Representative dotplots of Foxp3 and RORγ staining in mesenteric CD4+ T cells. Isotype controls were used to determine Foxp3 and RORγ-specific staining. **B-** The $CD4^+CD25^{high}$ population was considered as containing principally Treg when ≥90% of the population expressed Foxp3.

Table I: cell culture reagents and monoclonal antibodies

**Medium complet**	
RPMI 1640 with glutamax	Life Technologies
supplementes with 10% fetal calf serum	Life Technologies
streptomycin	Life Technologies
penicilin (100µg/ml)	Life Technologies
2% sodium pyruvate	Life Technologies
0.05 mM Beta-mercaptoethanol	Life Technologies
10 ml Hepes	Life Technologies
**Recombinant cytokines**	
IL-27 (rm)	R&D systems
IL-6 (rr)	R&D systems
IL-1 beta (rr)	R&D systems
IL-23 (rr)	R&D systems
TGF-beta (hr)	R&D systems
IL-27 (hm)	R&D systems
IL-2 (mr)	Immunotools
IL-2 (hm)	Miltenyui biotec
Il-27 (rr)	Curie Institut/ PX-therapeutics
**Purified Antibodies**	
Mouse anti-rat CD3	BD pharmingen
Mouse anti-rat CD28	BD pharmingen
Mouse anti-human CD28	BD pharmingen
Mouse anti-human CD3	Eurobio-Mabtech
**Conjugated Antibodies**	
anti-rat CD4	BD pharmingen
anti-rat IFN	BD pharmingen
anti-mouse ROR γt	BD pharmingen
anti-rat CD62L	eBioscience
anti-rat ICOS	eBioscience
anti-rat IL-17	eBioscience
anti-rat CD25	eBioscience
anti-mouse/rat TNF- α	eBioscience
**Neutralizing antibodies**	
anti-rat IFN- γ	BD pharmingen
anti-rat IL-4	BD pharmingen
anti-rat IL-10	BD pharmingen
**Phospho antibodies**	
STAT1 phosphorylated tyrosine 701	BD pharmingen
STAT3 phosphorylated tyrosine 705	BD pharmingen
**Other Reagents**	
Phorbol myristate acetate (PMA)	Sigma
Ionomycin (iono)	Sigma
Brefeldin A (BFA)	Sigma
Mytomycin	Sigma

Table II: SpA patient characteristics

Characteristic	SpA (n = 18)	Controls (n = 11)
Age: mean (range) in years	51 (28-72)	32 (22-47)
Sex, no (%) of males	11 (61)	7 (64)
HLA-B27+ (%)*	17 (94)	0 (0)
SpA subtype, no (%)		
AS	13 (72)	NA
Non-radiographic axial SpA	5 (28)	NA
BASDAI: mean (range)	3.4 (0-7.4)	NA
Disease duration: mean (range) in years	24,8 (11-48)	
Treatments, no (%) taking		
None	6 (33)	NA
NSAID	7 (39)	NA
Sulfasalazin	2 (11)	NA
Biotherapy **	8 (44)	NA

SpA: spondyloarthritis; AS: ankylosing spondylitis; BASDAI: Bath Ankylosing Spondylitis Disease Activity Index; NSAID: non-steroidal anti-inflammatory drug; NA: not applicable.
* HLA-B27 was undetermined in 1 healthy control
**Biotherapies: anti-TNFα (7); anti-IL-12/IL-23 (ustekinumab, 1)

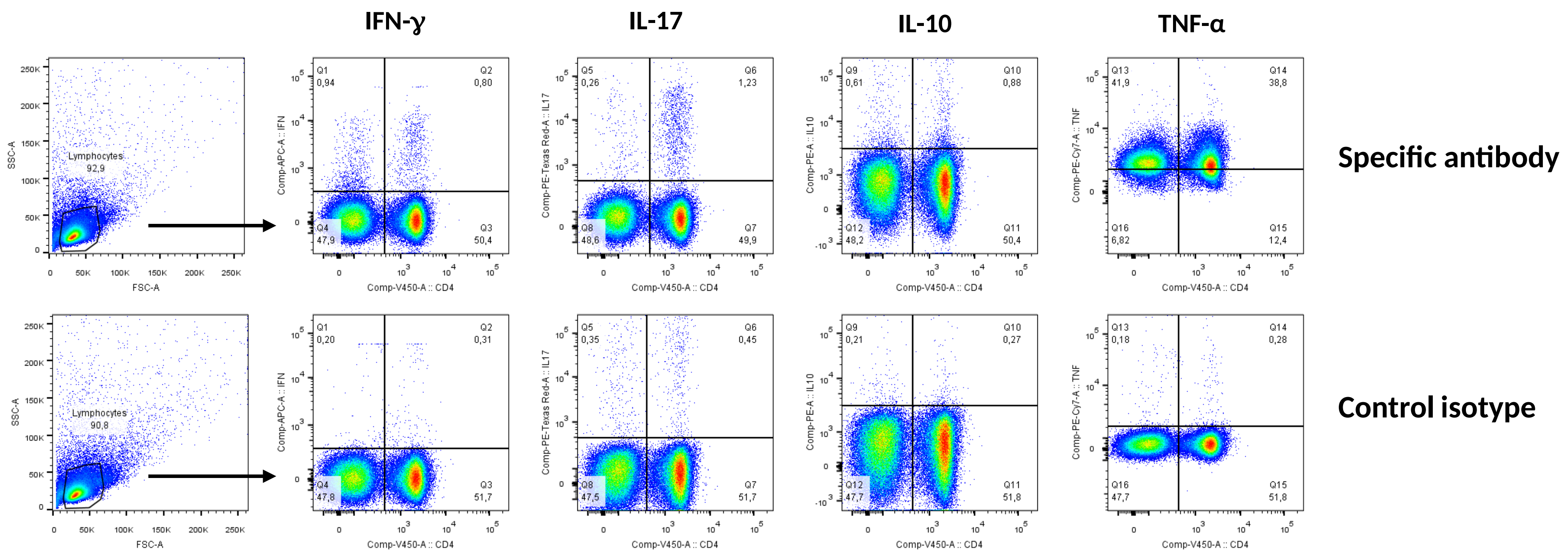

Supplementary Figure 3: Representative dot plots of intracellular cytokine staining of mesenteric CD4+ T cells. After 4 hours of stimulation with PMA, ionomycin in the presence of brefeldin A, total mesenteric lymph nodes cells were stained with anti-CD4 antibody and anti-cytokines specific antibodies or isotype control. To determine the percentage of cytokine-producing T cells, we subtracted staining intensity with isotype control to staining intensity with anticytokine antibody.

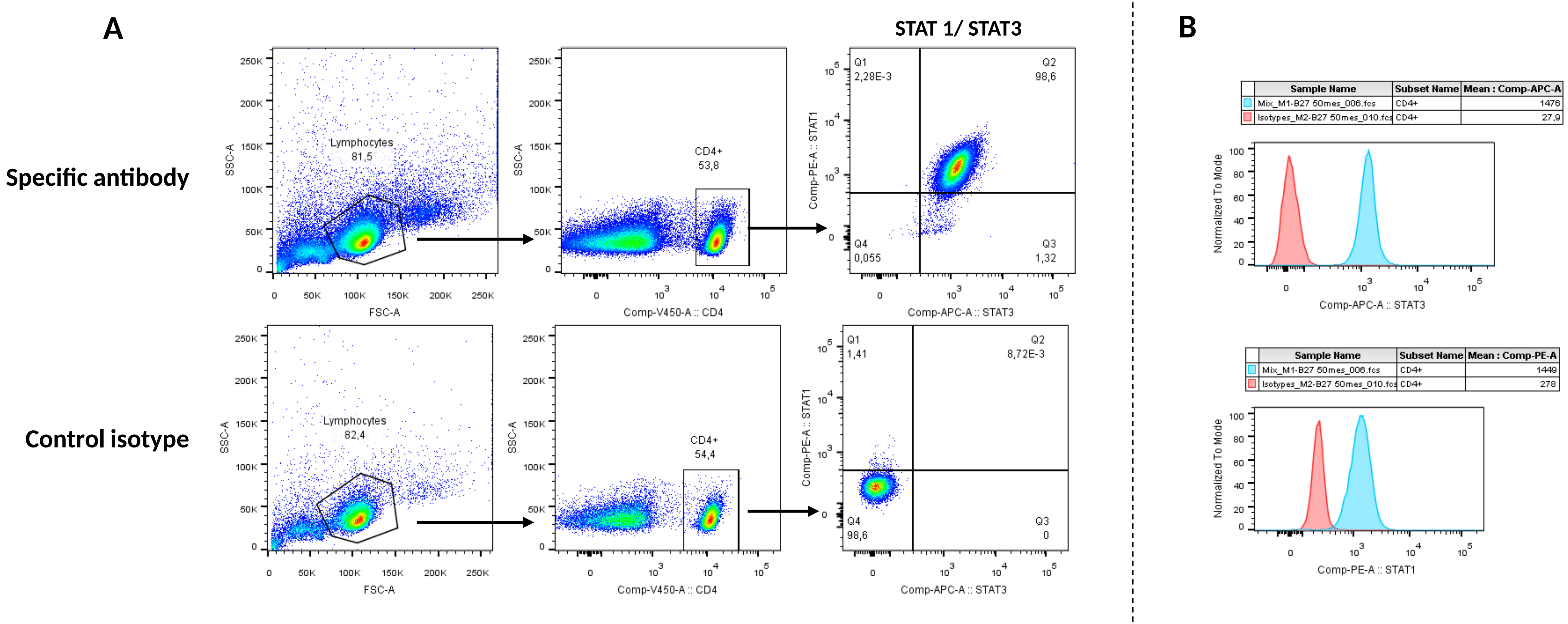

Supplementary Figure 4: **A-**Representative dotplots of STAT1 and STAT3 staining in mesenteric CD4+ T cells. To determine STAT1- and STAT3 -specific staining, we performed staining with respective isotypes control.
B- Histogram representing STAT1/ STAT3 and isotype control staining with fluorescence mean intensity (MFI) values .

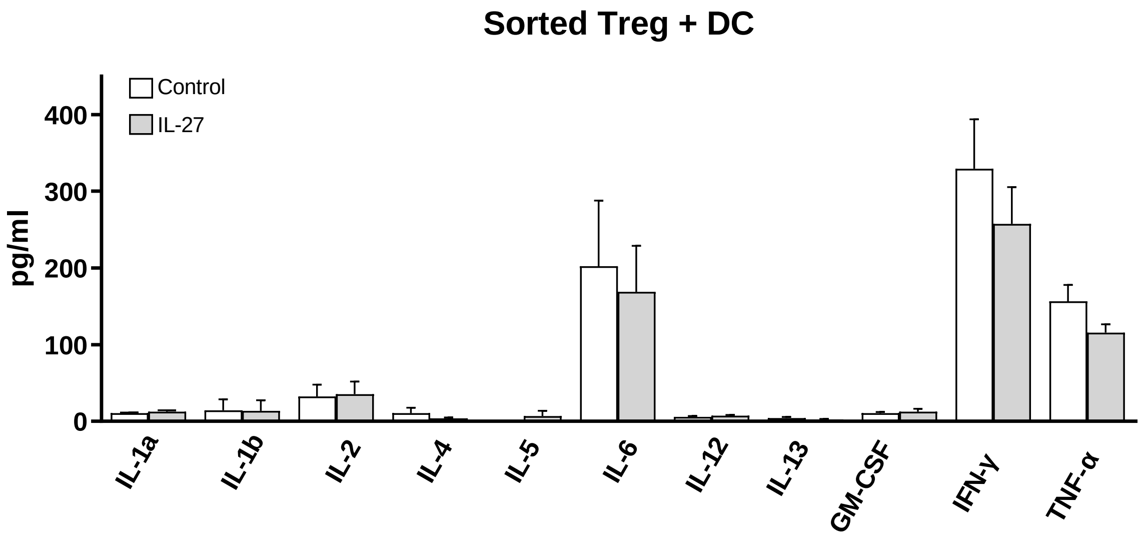

Supplementary. *F*igure 5: Cytokines production by B27-rat Treg cocultured with B27-rat DCs in the presence or absence of rmIL-27. A panel of 11 cytokines was studied, using multiplex ELISA assay in the supernatants of Treg and DCs cocultures. Values are the mean + SEM of 4 independent experiments.

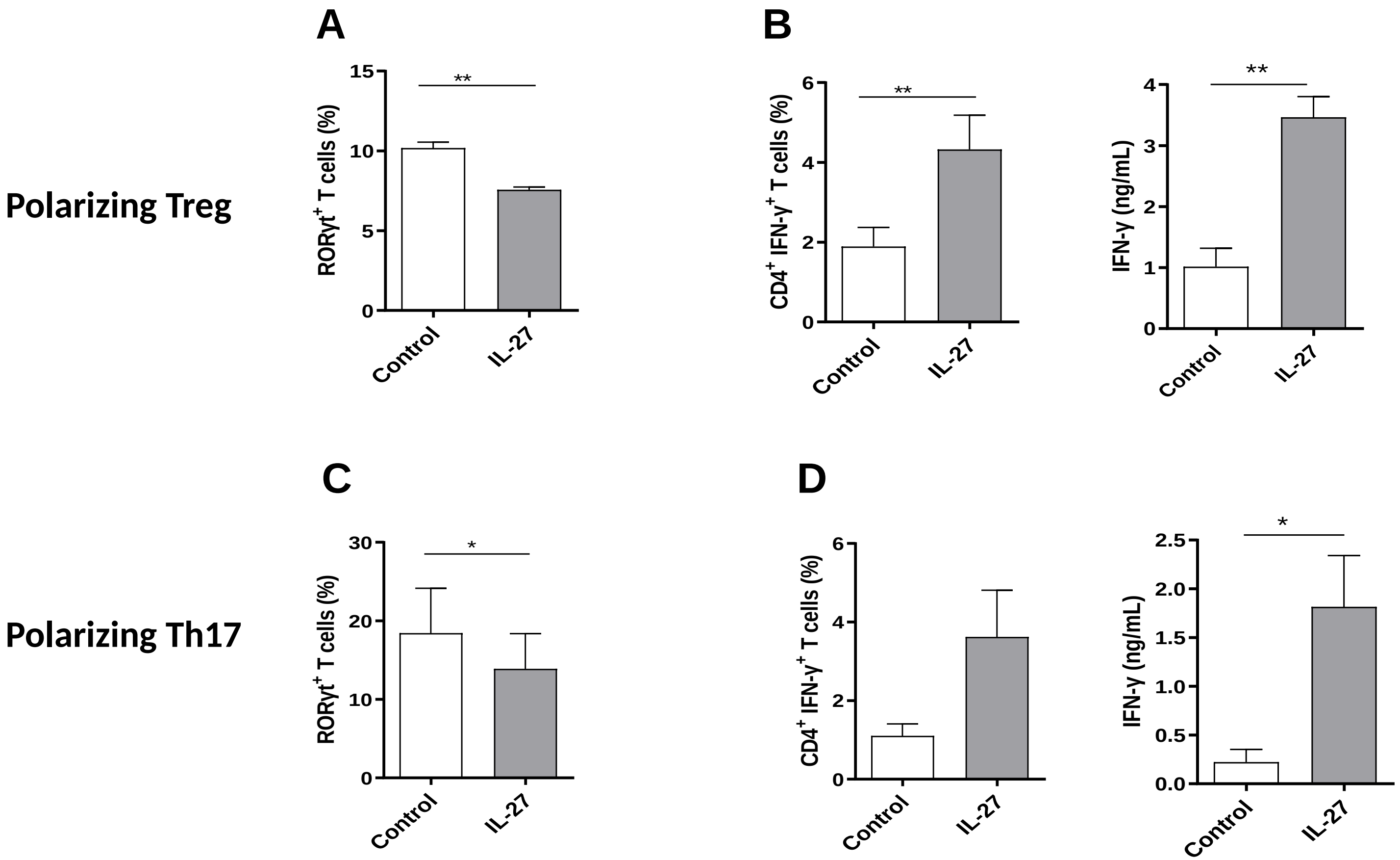

Supplementary. Figure. 6: IL-27 downregulates RORgt and upregulates IFN-γ in Treg and Th$_{17}$ polarizing CD4$^+$ LN T cells from B27-rat. CD4$^+$ CD25$^-$ CD62L$^+$ naïve T cells were polarized *in vitro* by coculture with CD103$^+$ DCs in (A and B) Treg or (C and D) Th$_{17}$ conditions during 6 days in the presence or absence of IL-27. The frequency of RORγt$^+$ T cells was determined by flow cytometry and IFN-γ level was evaluated after PMA-ionomycin stimulation by flow cytometry or ELISA in the supernatants. Values are the mean + SEM of ≥ 3 independent experiments. ** $p < 0.01$

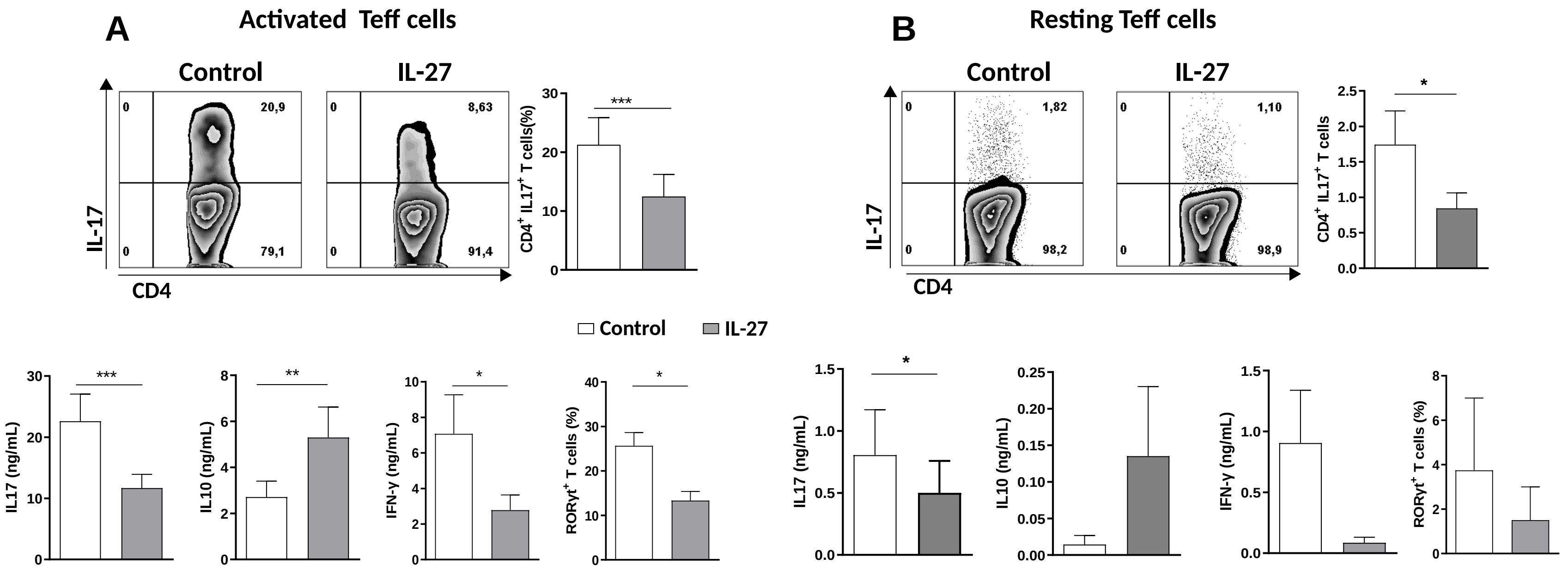

Supplementary. Figure. 7: Anti-inflammatory effect of IL-27 on activated and resting Teff cells in the absence of DCs. (A) Representative data of activated $CD4^+$ $CD25^{low}$ Teff cells from B27-rat stimulated for 3 days with anti-CD3/anti-CD28 in the presence or absence of IL-27 and restimulated with PMA-ionomycin-BFA. IL-17, IL-10 and IFN-γ levels were evaluated by ELISA in the supernatants at the end of the culture and $ROR\gamma t^+$ T cells frequency by flow cytometry. Values are the mean ± SEM of 3-7 independent experiments. ** $p< 0.01$, *** $p< 0.001$. (B) Representative data of resting $CD4^+$ $CD25^-$ $CD62L^-$ Teff cells from B27 rat stimulated for 3 days with anti-CD3/anti-CD28 in the presence or absence of IL-27 and restimulated with PMA-ionomycin-BFA. IL-17, IL-10 and IFN-γ levels were evaluated by ELISA in the supernatants at the end of the culture and $ROR\gamma t^+$ T cells frequency by flow cytometry. Values are the mean + SEM of 3-7 independent experiments. *$p<0.05$

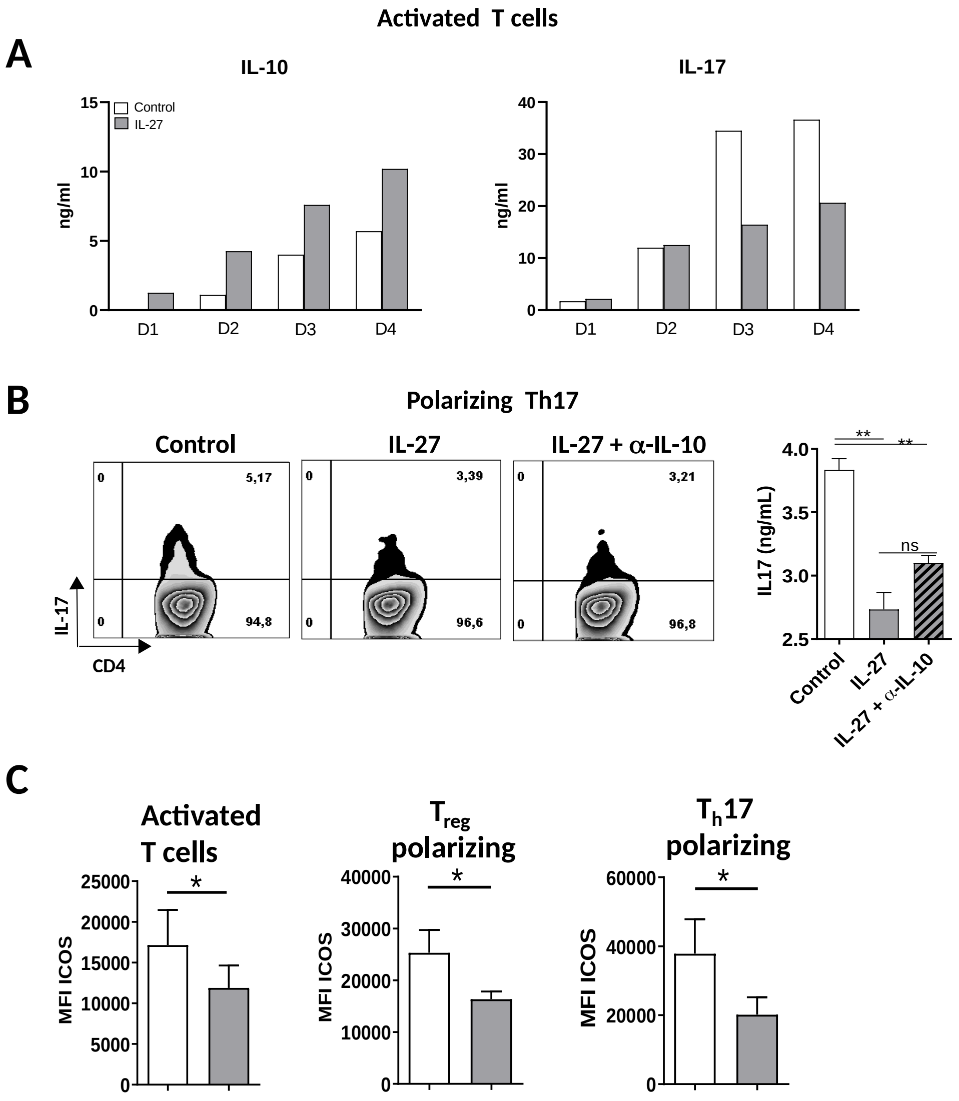

Supplementary. Figure. 8: Decreased IL-17 production induced by IL-27 is IL-10- and ICOS-independent. (A) kinetics of IL-27 effect on CD4$^+$CD25$^+$activated T cells from mesenteric LN of B27-rat, the IL-17 and IL-10 levels were determined in culture supernatants by ELISA. One representative experiment from two. (B) Left panel: representative dot-plot of IL-17$^+$ cells among B27-rat CD4$^+$ T cells cultured during 6 days in Th$_{17}$-polarizing conditions with coated anti-CD3 and soluble anti-CD28 in the presence or not of IL-27 and anti-IL-10 blocking antibody, starting from naïve CD4$^+$ T cells. Right panel: level of IL-17 in culture supernatants. (C) ICOS expression on cultured activated T cells, Treg-polarizing, and Th$_{17}$-polarizing cells. Values are the mean + SEM of 3 independent experiments. ** $p<0.01$; * $p<0.05$; ns = not significant.

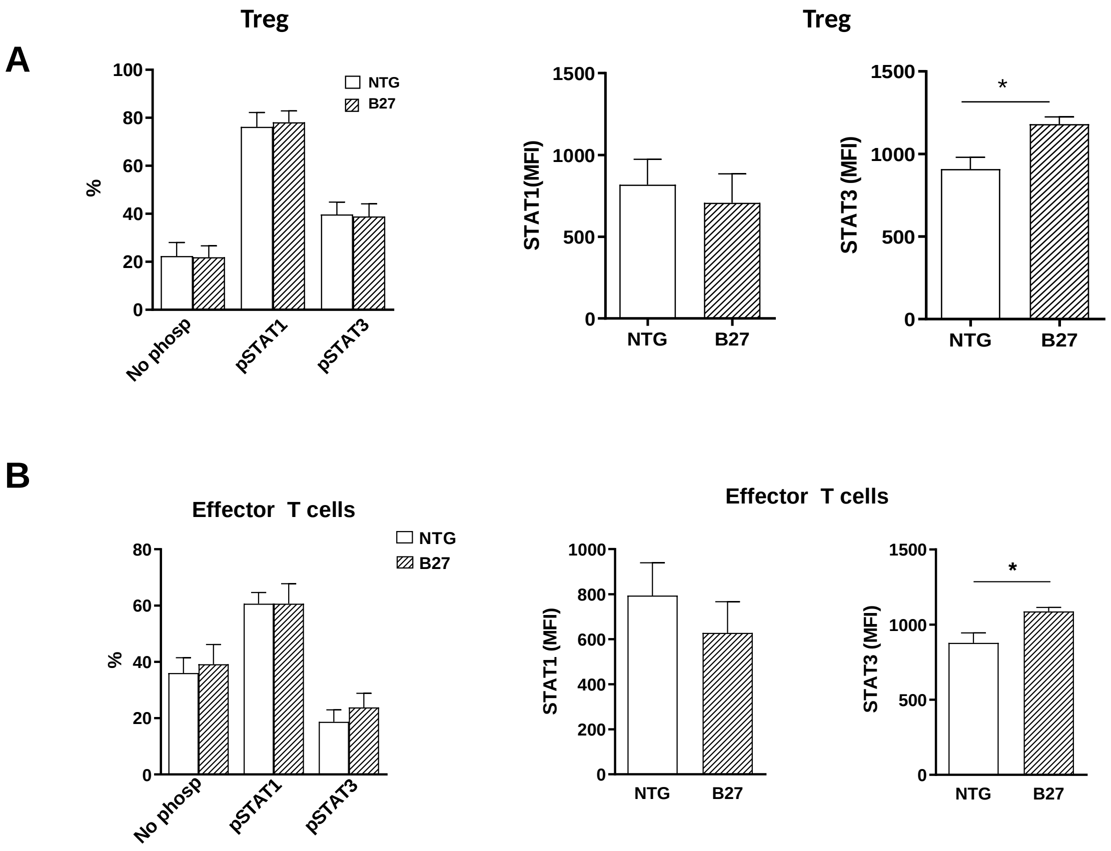

Supplementary. Figure. 9: IL-27 induces similar phosphorylation of STAT1 and STAT3 in NTG and B27-rat despite increased STAT3 level in B27-rat T cells. (A) Percentage of STAT1 and STAT3 phosphorylation after IL-27 stimulation in Treg ($CD4^+$ $CD25^{high}$) and Teff ($CD4^+$ $CD25^-$) cells from NTG and B27-rat LN. Values are the mean + SEM of 6-7 independent experiments. (B) Total STAT1 and STAT3 levels were analyzed by flow cytometry in gated activated $CD4^+$ $CD25^+$ T cells and $CD4^+CD25^-$ Teff cells from NTG and B27-rat LN. Values are the mean + SEM of 3 independent experiments. $^*p<0.05$.

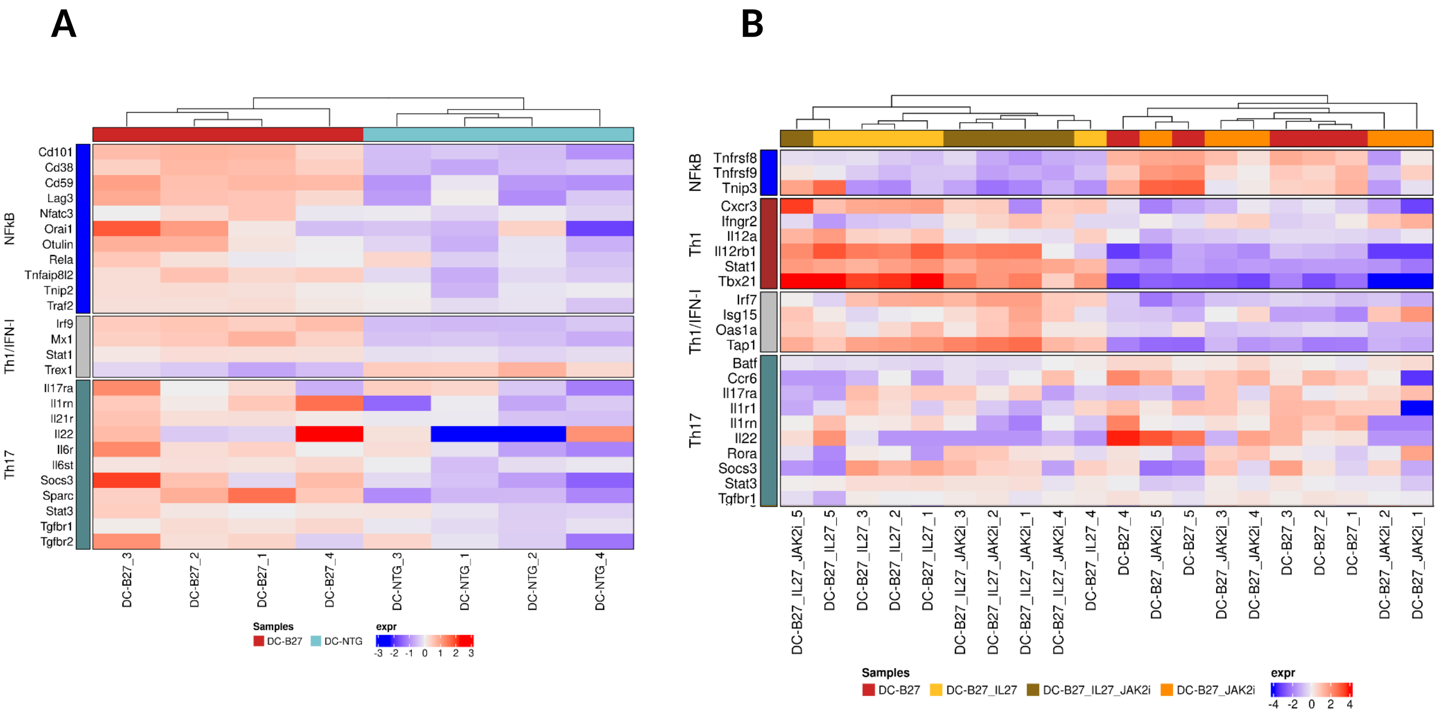

Supplementary. Figure. 10: RNAseq analysis of naïves CD4⁺ T cells from B27 rat after coculture with cDC2 from NTG or B27 rats in the presence or not of IL-27 and AG490, a JAK2 inhibitor: RNA from naive CD4⁺ CD25⁻ CD62L⁺ T cells from B27-rats sorted after 1 day of coculture with NTG or B27-rat cDC (CD103⁺ CD4⁺) or, in the presence or not of IL-27 or JAK2 inhibitor (JAK2i, AG390), was isolated and global gene expression was determined by RNA-seq. (A) Heatmap of normalized transcript counts of genes of interest comparing cocultures with DCs from either NTG or B27-rats. (B) Heatmap clustering (same experiment as Figure 4) of normalized transcript counts of genes of interest, comparing cocultures with B27-rat cDC2 (CD103⁺ CD4⁺) in the presence or not of IL-27, JAK2i or IL-27 + JAK2i.

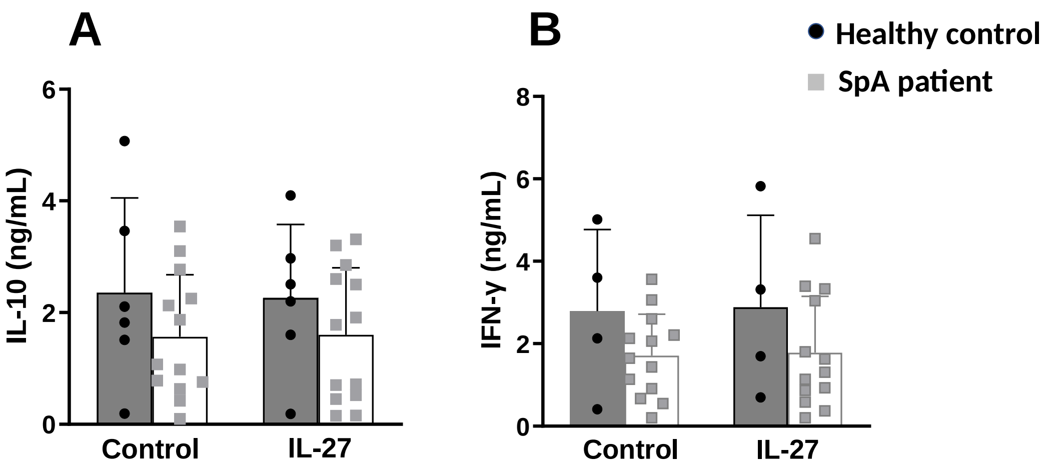

Supplementary. Figure. 11: Effect of IL-27 on IL-10 and IFN-g production by peripheral blood CD4^{+} T cells from SpA patients and healthy controls. (A) IL-10 and (B) IFN-g production by CD4^{+} T cells from SpA patient cultured during 6 days with IL-2 in the presence or absence of IL-27 was measured in supernatants by ELISA. Values are the mean + SEM of 6-13 individuals/group.

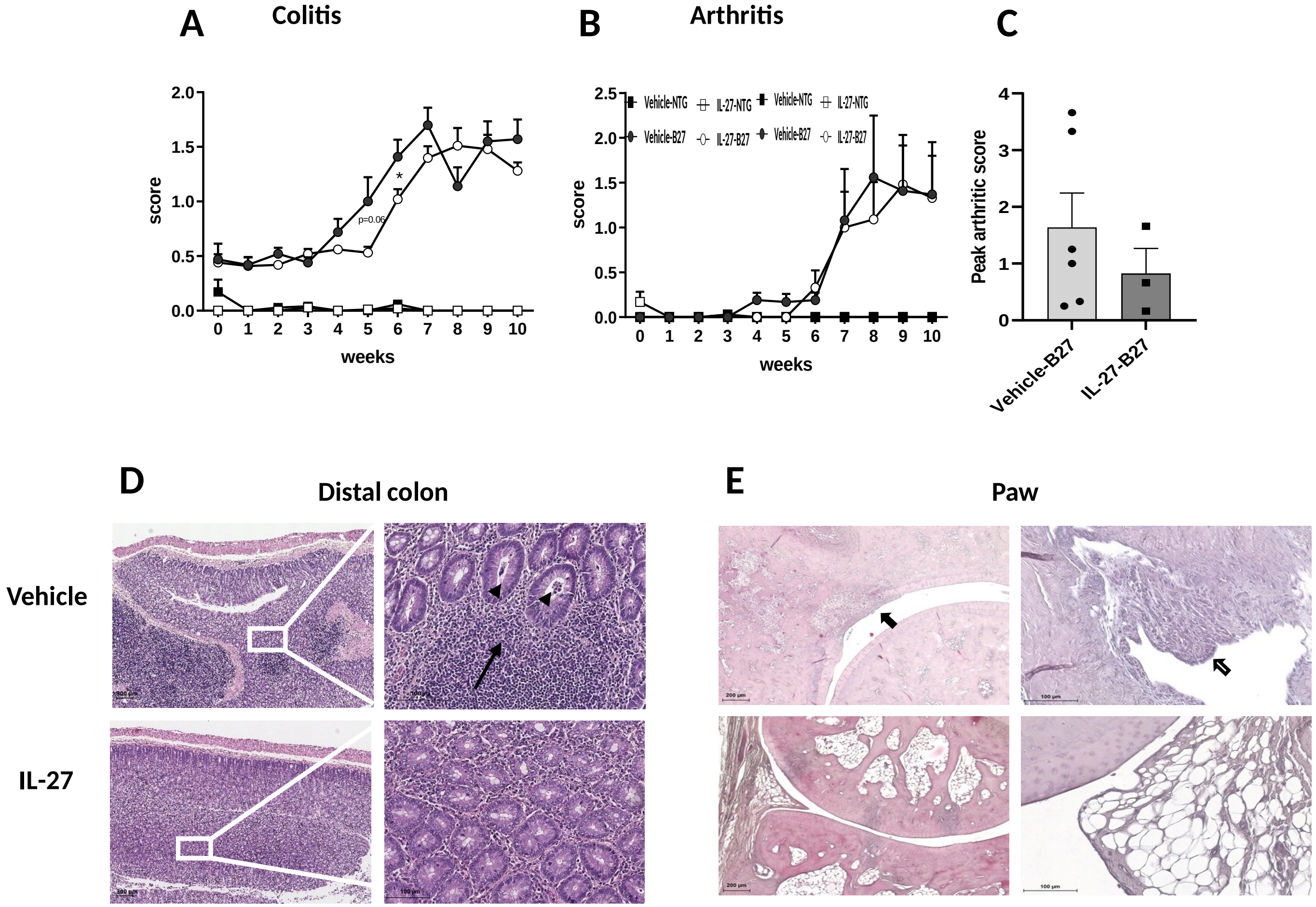

Supplementary. figure. 12: *In vivo* effect of rrIL-27 on SpA development in B27-rat. (A,B) Clinical score of (A) colitis and (B) arthritis from NTG and B27-rats treated three times per week with vehicle or rrIL-27, during 10 weeks (experiment 1). Values are the mean + SEM of 6-8 rats/group. * $p < 0.05$. (C) Peak arthritic score of B27-rats treated daily with vehicle or rrIL-27, during 12 weeks (experiment 2; mean + SEM). Representative examples of (D) distal colon and (E) joint (4 distinct paws) histology of B27-rats treated with vehicle or rrIL-27 daily, during 12 weeks (experiment 2). (D) Left panels: original magnification x 40. Right panels: selected areas are shown at higher magnification. Arrow: infiltration of the *lamina propria* by inflammatory cells. Arrowheads: crypts abscess (E) Cartilage and bone erosion close to synovitis (close arrow) and synovial hyperplasia with inflammatory cell infiltrate (open arrow) in B27-rats treated with vehicle (top panels). Normal aspect of synovial tissue, bone and cartilage in B27-rats treated with IL-27 (bottom panels).
